# Performance and Impact of Crosslinking Level of Hierarchical Anion-Exchange Membranes on Demineralization of a Complex Food Solution by Electrodialysis

**DOI:** 10.3390/membranes14070155

**Published:** 2024-07-12

**Authors:** Elodie Khetsomphou, Francesco Deboli, Mateusz L. Donten, Laurent Bazinet

**Affiliations:** 1 Institute of Nutrition and Functional Foods (INAF), Dairy Science and Technology Research Centre (STELA) and Department of Food Sciences, Université Laval, Quebec, QC G1V 0A6, Canada; elodie.khetsomphou.1@ulaval.ca; 2 Laboratoire de Transformation Alimentaire et Procédés ÉlectroMembranaires (LTAPEM, Laboratory of Food Processing and ElectroMembrane Processes), Université Laval, Quebec, QC G1V 0A6, Canada; 3 Department of Chemical Engineering, KU Leuven, 3001 Leuven, Belgium; debolifrancesco@gmail.com; 4 Amer-Sil SA, 8281 Kehlen, Luxembourg; mateusz.donten@gmail.com

**Keywords:** electrodialysis, whey, demineralization, hierarchical anion-exchange membrane

## Abstract

Promising results were recently reported for hierarchical ion-exchange membranes, fabricated by the UV crosslinking of a thin functional coating on a porous substrate, on model NaCl solution demineralization by electrodialysis (ED). Hierarchical anion-exchange membranes (hAEMs) have never been tested with complex solutions to demonstrate their potential use in the biofood industry. The impact of three different crosslinking densities of the ion-exchange coating (EbN-1, EbN-2 and EbN-3) on the performances of whey demineralization by ED was investigated and compared with commercial AMX. The results showed that by increasing the coating crosslinking density, the membrane conductivity decreased, leading to an increase in the global system resistance during whey demineralization (from +28% to +64%). However, 18% sweet whey solutions were successfully treated until 70% demineralization for all membranes. The energy consumption (averaged EbN value of 14.8 vs. 15.1 Wh for AMX) and current efficiency (26.0 vs. 27.4%) were similar to the control. Potential fouling by non-protein nitrogen was detected by ATR-FTIR for hAEMs impacting some membranes properties and ED performances. Overall, EbN-1 obtained results were comparable with the benchmark and can be considered as an alternative membrane for whey demineralization by ED and other applications in the demineralization of complex products from the food industry.

## 1. Introduction

Liquid by-products and wastewater are main sources of concerns for many different industrial fields like chemical and agri-food sectors [1,2,3,4]. In the past years, ED has proven its potential and eco-efficiency to manage waste and by-products resources [4,5]. Indeed, ED separates charged molecules through selective ion-exchange membranes (IEMs) under the effect of an electric field [6,7,8]. The share represented by IEMs in a fully automated ED unit amounts to 25–30% of the total cost [9]. Innovation in membrane technology can make this system even more competitive and economically feasible for lower value applications. 

Hierarchical ion-exchange membranes (hIEMs) for electrodialysis (ED) were recently developed to improve performance of ion-exchange membranes as well as to reduce their fabrication cost. These membranes were first introduced by coating a porous filtration membrane substrate with a thin layer of amino-functionalized anion-exchange material [10]. Tested on NaCl and food solutions for demineralization by ED, the obtained results were promising. Lately, the newest improvement of such membranes was made in the coating formulation. Deboli et al. developed new formulations using commodity precursors and crosslinking by the UV-initiated polymerization of the ion-exchange coating [11]. This fabrication process offers mechanical stability from the porous substrate as well as the selectivity of the ion transport controlled by the dense ion-exchange coating. The advantages of this concept reside in decoupling the required mechanical stability and ion selectivity in two different matrices. Moreover, the used of commodity precursors and the fabrication process of these membranes were envisioned to allow an easy industrial production scale up. 

Recently, the latest hierarchical cation-exchange membranes (hCEMs) were tested for both demineralization by the ED of model NaCl [11] and real food solutions such as sweet whey [12]. Indeed, sweet whey is a major by-product of cheesemaking and also the most ED-treated solution, every year, in the dairy industry [13,14,15]. The demineralized whey solutions are commonly used in food formulations and, for instance, for infant formula, for which a high demineralization rate is required [16]. Khetsomphou et al. were found that the best hCEM formulations had similar performances to the commercial reference [12]. A common ED system is composed of both CEMs and AEMs, but all of the previous studies were carried out solely on hCEMs. In addition, no studies can be found on hierarchical anion-exchange membranes (hAEMs) for whey demineralization by ED in the literature. Moreover, no fouling was reported for hCEMs in these previous studies. However, knowing that AEMs are generally more prone to fouling [17,18,19,20] and that fouling/scaling is a major concern for the use of IEMs for biofood applications [21,22,23,24], investigation needs to be carried-out on hAEMs. 

This paper presents hAEM with three formulations of the yielding to three different crosslinking densities of the ionomer. The objectives of this work are (1) to demonstrate their feasibility for whey demineralization of complex solutions like food steams used in the industry, (2) to study the performances of these hAEMs stacked in an ED cell for whey demineralization, (3) to study the effect of the crosslinking density on membrane performances, (4) to evaluate and to identify potential fouling, and (5) to evaluate these performances in comparison with those of a food grade homogeneous anion exchange membrane largely used in the food industry.

## 2. Materials and Methods

### 2.1. Materials

**Figure 1:**
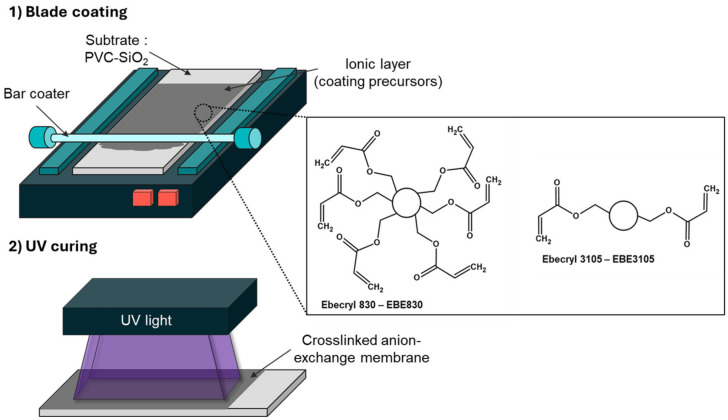
Fabrication process of the hierarchical anion-exchange membranes.

Amer-Sil S.A. (Kehlen, Luxembourg) produced and provided the hAEMs. The membranes and EbN coating chemistry consist of a commercially available acrylic resin polymerized with acrylic monomers containing ammonium groups. The coating was applied on top of a PVC-SiO_2_ porous substrate membrane produced by Amer-Sil. The fabrication process was first reported and detailed by Deboli et al. [11]. Three different coating formulations were prepared for this study and Table 1 reports the formulation details. The ratios of oligomers and monomers were the same as those used by Deboli et al. for demineralization of model solutions of salts in experiments comparing their performance with a heterogeneous membrane [11], known as less efficient than homogeneous membranes used in this study [12]. A schematic of the membrane fabrication process is detailed in Figure 1.

Neosepta food grade ion-exchange membranes were bought from Astom (Tokyo, Japan): an anion-exchange membrane (AMX), used as a benchmark, and a cation-exchange membrane (CMX). These membranes are some of the best performing industrial homogeneous membranes for food applications.

Parmalat (Victoriaville, QC, Canada) provided the sweet whey powder (Lot: 1019003001). The same lot of sweet whey powder was used for all experiments. The approximate composition of the whey powder provided by the manufacturer was protein (11%), lactose (68%), and moisture (5%). The ash content as well as the composition in the main cations (7%—Cations: K^+^ (55.6%), Na^+^ (19.9%), Ca^2+^ (19.9%) and Mg^2+^ (4.6%)) were previously determined by Khetsomphou et al. [12].

### 2.2. Methods

#### 2.2.1. Characterization of Membrane and Morphology

The physicochemical properties of the membranes were characterized before experiments in an ED cell.

##### Thickness

A digital micrometer from Marathon Watch Company LTD. (Richmond Hill, ON, Canada) was employed to measure the thickness of hAEMs and AMX benchmark according to Kadel et al. [25].

##### Microscopy

The homogeneity and continuity of the ion-exchange coating layer of the hAEMs were assessed by digital microscopy (VHX-7000, Keyence, Japan). Cross sections of the membranes were used to evaluate the coating thickness using digital image analysis (VHX-7000 Ver 1.4.23.17, Keyence software, Osaka, Japan).

##### Roughness

The roughness of the membrane was determined using a surface profilometer (DektakXT, Bruker Nano Surfaces, Tucson, AZ, USA) as described by Kadel et al. [25]. Same parameters were used as Khetsomphou et al. [12]. The scan area was 1000 µm × 1000 µm per measure. The analysis software used was Vision64 (Bruker Nano Surfaces, Tucson, AZ, USA). The coating layer topography of the membranes was assessed by measuring the average values of the highest heights and deepest alleys (Rz) and the average roughness (Ra).

##### Contact Angle

The contact angle value of all membranes was measured with a goniometer Theta OneAttension (Biolin Scientific, Linthicum Heights, MD, USA) by sensile drop method described in details by Kadel et al. [25]. 2 µL of distilled water drop was dispensed on the membrane surface. The drop profile was analyzed by OneAttension software (Version 2.4, Biolin Scientific, Espoo, Finland). 

##### Ion-Exchange Capacity

The ion-exchange capacity (IEC) of all membranes was evaluated using the Mohr titration method [26]. The membranes were soaked in an HCl (1 N) solution overnight to convert them into Cl^−^ form. The membranes were rinsed with distilled water to remove excessive Cl^−^ ions. Afterwards, the membranes were immersed for one hour in a 50 mL solution of Na_2_SO_4_ (0.25 N). The titration of the released chloride ions was performed with AgNO_3_ (0.05 N) using K_2_CrO_4_ as an indicator. The membrane IEC value was calculated with the following equation:

(1) $$IEC=\frac{{[AgNO}_{3}]V_{{AgNO}_{3}}}{m_{dry\;membrane}}$$

with IEC as the ion-exchange capacity expressed in meq.g^−1^ of dry membrane, ${[AgNO}_{3}]$ and $V_{{AgNO}_{3}}$ as the concentration (N) and volume of AgNO_3_ (mL) used, respectively, for the titration and $m_{dry\;membrane}$ as the mass of dry membrane (g). In the case of hAEMs, the mass of dry membranes includes the porous substrate and the IEM coating.

##### Conductivity and Ionic Conductance

The membranes’ ionic conductance and conductivity were determined in a 0.5 M NaCl solution using a designed clip from the Laboratoire des Matériaux Echangeurs d’Ions (Université Paris XII, Créteil, France) as described by Khetsomphou et al. [12]. The electrical resistance (R) was calculated from the measurement of the electrical conductance (G) (G = 1/R). The membrane transversal electrical resistance, Rm (in Ω), was determined using the following equation:

(2) $$R_{m}=R_{m+s}-R_{s}$$

where $R_{m+s}$ represents the overall resistance (both membrane and reference solution measured in Ω) and $R_{s}$ corresponds to the reference solution’s resistance (in Ω).

The electrical conductivity (κ, in S.cm^−1^) can be calculated using Equation (3) [27,28]:

(3) $$\kappa =\frac{l}{R_{m}A}$$

where l represents the thickness of the membrane (in cm), and *A* is the area of the measure (1 cm^2^ in this study setup).

##### Selectivity

The selectivity of the membranes was calculated by measuring the potential of the membranes with a potentiostat (Origaflex 01A, Origalys, Rillieux-la-Pape, France) as described by Deboli et al. [29]. The membrane is placed between laboratory made two-cell compartments equipped with, in each half cell, a saturated calomel electrode. One compartment is filled with 0.1 M KCl solution and the other compartment with 0.5 M KCl solution. The selectivity of the membranes was calculated with the following equation:

(4) $$\alpha =\frac{∆V_{exp}}{∆V_{th}}\times 100$$

where $∆V_{exp\;}$ represents the experimental potential (in mV), and $∆V_{th}$ the theoretical potential (36.68 mV in this study [11]).

#### 2.2.2. Electrodialysis Experiments

##### ED Set-Up and Configuration

**Figure 2:**
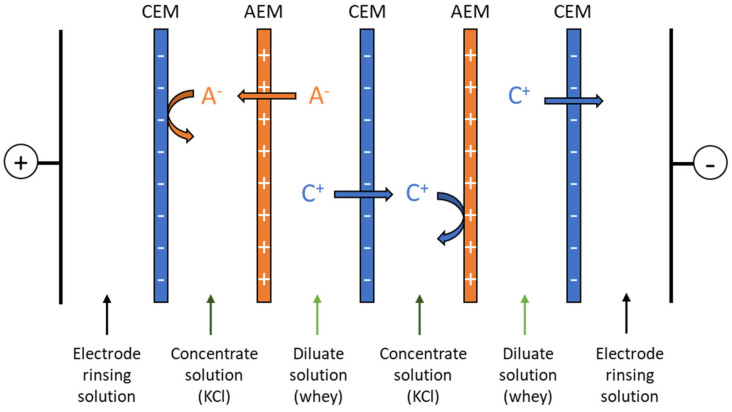
Electrodialysis cell configuration employed for whey demineralization. A^−^: anion; C^+^: cation; CEM: cation-exchange membrane and AEM: anion-exchange membrane.

An ED MP-type cell (ElectroCell AB, Täby, Sweden) was used for ED experiments. The effective membrane surface area was 100 cm^2^. The ED configuration consisted of two hAEMs or benchmark food grade AMX along with three Neosepta food grade cation-exchange membranes (CMX). Figure 2 shows the arrangement of the stack that has been placed to obtain a six-compartment cell configuration. The coating side of the formulated hAEMs was facing the diluate compartment. The membranes were activated by conditioning overnight in 2 g.L^−1^ KCl solution. A total of 20 g.L^−1^ of Na_2_SO_4_ (0.8 L) was used as rinsing solution, 2 g.L^−1^ of KCl solution (0.6 L) was used as concentrate and sweet whey (0.6 L) composed the diluate for all of the experiments. For the anode and cathode, a dimensionally stable electrode (DSA-O_2_) and a stainless-steel electrode cathode were used, respectively. Pumps from the Baldor Electric Company (Fort Smith, AR, USA) were operated to recirculate and promote a turbulent flow in each compartment. Flow rates were controlled by flowmeters (Blue-White Industries Ltd., San Diego, CA, USA). The linear velocity along the membrane was 0.12 cm.s^−1^. The system was operated in voltage-controlled mode and 13.0 V were imposed on the system for all demineralization. This value was chosen from previous experiments to ensure a better comparison of the system performances [12]. 

##### Limiting Current Density

The system’s limiting current density in real conditions was evaluated for each membrane in duplicate. For the repetitions, the same membranes were used after rinsing with water between the two runs. To recover, the system was left to rest for 1 h to avoid impacting the integrity of the membrane [12]. The related voltage was also calculated. The system was tested by using the configuration described beforehand and by gradually increasing the voltage by 0.5 V increases from 0 to 30 V. Throughout the experiment, the current intensity was recorded. The limiting current densities were determined by the Cowan and Brown method [30]. 

##### Performance Evaluation

pH

The pH of KCl and sweet whey solutions was measured using a pH meter (Models SympHony SP70P and SympHony SP20, VWR Scientific Products, Radnor, PA, USA).

Conductivity

KCl and whey solution conductivities were determined with a conductivity meter (Model 3100, Yellow Spring Instruments, Yellow Springs, OH, USA) equipped with an immersion probe (Model 3252, cell constant K = 1 cm^−1^) and an automatic temperature compensation (ATC).

Demineralization

The rate of demineralization (in %) was calculated throughout the experiment with the following equation:

(5) $$DR=\frac{\delta_{0}-\delta_{t}}{\delta_{0}}\times 100$$

with $\delta_{0}$ and $\delta_{t}$ as the conductivity values of the diluate at time 0 and t (when the process was stopped), respectively.

Mineral Concentration

During ED treatments, KCl compartment fractions were collected every 15 min to measure the mineral concentration throughout the experiment. Potassium, magnesium, calcium, sodium and phosphorus ions were analyzed using ICP-OES (Agilent 5110 SVDV Agilent Technologies, Victoria, Australia) as described by Khetsomphou et al. [12]. Chloride ions concentration analysis was performed using a Quikchem 8500 flow injection analyzer (serie 2, Zellweger Analytic, inc., Milwaukee, WI, USA) according to the Quikchem method 10-117-07-1-C: chlorine in water [12]. 

##### Current Efficiency and Energy Consumption

After each experiment, current efficiency (*η*) and energy consumption (*EC*) have been evaluated according to the following equations [29]:

(6) $$\eta =\frac{zFV_{d}\left(C_{t}-C_{0}\right)}{n\int_{0}^{t}I\left(t\right)dt}$$

(7) $$EC=U\int_{0}^{t}I\left(t\right)dt\;$$

where z  is the absolute valence (no unit) of the transported ion, *F* is the Faraday constant (C.mol^−1^), $C_{t}$ and $C_{0}$ are the diluate whey concentrations at times t and 0 (in mol.m^−3^), $V_{d}$ is the diluate solution volume (in m^3^), n is the number of cell pairs, *I* is the current (in A), *U* is the voltage (in V) and t is the process duration (in h).

##### Whey Demineralization

An 18% whey solution was used for all whey demineralization experiments as the dairy industry commonly uses this concentration. The ED experiments were performed in triplicates.

The system was operated in voltage-controlled mode to have constant electric field strength. The objective for each experiment was to reach a 70% whey demineralization rate [12]. For the repetitions, the same membranes were used for each specific membrane type. The system was disassembled after the three runs so that the impact on the membranes of consecutive runs could also be evaluated. Having consecutive runs also allows for an evaluation of whether fouling is present or not [12]. Between each ED run, to avoid the contamination of feed and electrolyte solutions from one ED run to another ED run, the system was cleaned with distilled water. A flow rate of 0.7 L.min^−1^ for concentrate and diluate compartments was set, and a flow rate of 1.0 L.min^−1^ was used for the electrode rinsing solution compartments [12]. Experimental parameters like the current across the ED stack, conductivity and pH of the diluate solution were recorded every 15 min throughout the experiment. DR, current efficiency and energy consumption were analyzed for the global performance of the membrane in ED. 

Finally, each membrane was evaluated for its contact angle, thickness and conductivity after the third run. 

#### 2.2.3. Fouling Assessment

As mentioned previously, anion-exchange membranes are more prone to fouling, and consequently, membrane properties can be changed after ED when fouling appears. Hence, different methods, complementary to membrane characterization and morphology, were used to identify the nature of potential fouling (organic fouling or scaling) on the membrane surfaces.

##### SEM-EDS

Images of the membrane surfaces in contact with the diluate solution were taken by scanning electron microscopy (Quanta 3D FEG, Hillsboro, OR, USA). The microscope was equipped with an energy dispersive spectrometer (EDX) detector (PV8206/60 Genesis XM2, EDAX, Tokyo, Japan) and elemental analyses were performed. The EDS conditions were a working distance of 10.0 mm and accelerating voltage of 10 kV. Sodium, potassium, calcium, chlorine, phosphorus, sulfur, oxygen, and carbon were elements of interest [31].

##### ATR-FTIR

The surface of the membrane in contact with the feed solution was analyzed by attenuated total reflection–Fourier transform infrared (ATR-FTIR) to detect the presence or absence of organic fouling. The membranes were dried 5 days in a desiccator prior to analysis. The membranes were analyzed in a spectrometer from Nicolet Instrument Corp. (Madison, WI, USA) over a zinc selenide ATR crystal. A 30 min delay to the system was allowed to dehumidify the optical bench. The spectrum of vapor was subtracted from the original spectra to establish the baseline [31]. The spectra were smoothed in accordance with the method of Ayala-Bribiesca et al. [31].

#### 2.2.4. Statistical Analyses

The experiments for current–voltage curves were performed in duplicate. One-way analyses of variance (ANOVA) and Tukey tests at a probability level of 0.05 were used for statistical analysis. The Systat SigmaPlot software (Version 12.0) was used for data treatments. Triplicates were conducted on all ED experiments and the data were subjected to ANOVA, Tukey (among all of the membranes) and Dunnett (AMX as the reference) tests at a probability level of 0.05. A *t*-test at a probability level of 0.05 was performed to compare the thickness, contact angle and conductivity before and after the ED testing of each membrane. 

## 3. Results

### 3.1. Characterization of Membrane and Morphology

Within the fabricated membranes, the membrane thickness (0.419 ± 0.012 mm) showed no significant difference, whereas EbN-3 exhibited a higher coating thickness compared to EbN-1 (54.34 ± 2.67 µm vs. 36.59 ± 4.41 µm, respectively) (Table 2). Overall, because of the substrate of the hAEMs, these membranes were thicker than the reference (+74%).

**Figure 3:**
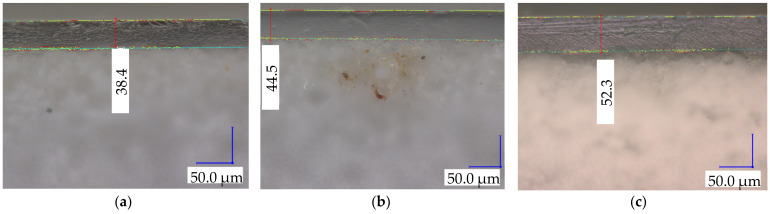
Cross-section micrographs for (**a**) EbN-1, (**b**) EbN-2 and (**c**) EbN-3.

Following the cross-section micrographs of the fabricated membranes, the layered hierarchical structure of all the hAEMs was demonstrated and observed by the sharp interface between the coating layer and the substrate (Figure 3). The images in Figure 3 also demonstrated no sign of defects (pits or cracks).

Ra values were the same for all tested membranes with an averaged value of 0.42 ± 0.15 µm (Table 2). Regarding Rz values, the statistical analysis revealed that EbN-2 and EbN-3 presented significantly higher Rz values (Table 2). The cited membranes may present higher rugosity than EbN-1 and the reference. Overall, the values were small, and the membranes can be considered smooth. Large standard deviations can be noticed, which are due to the small value reported, the homogeneity of the surface of the membrane and the probe’s sensitivity. Similar observations were previously reported for such a type of membrane with a similar value order [12,25].

The selectivity of the fabricated membranes (Table 2) demonstrated an increase with increasing crosslinking density (EbN-1: 86% and EbN-3: 94%). Similar results were obtained by Khetsomphou et al. [12] for hierarchical cation-exchange membranes. Deboli et al. demonstrated that increasing the crosslinking density of the ion-exchange layer allows for an improvement in selectivity but at a cost of higher resistance. The initial ionic conductivity of the membranes is also displayed in Table 2. Within the fabricated hAEMs, EbN-2 and EbN-3 demonstrated a lower conductivity compared to EbN-1. The trend seems to indicate a decrease in conductivity with increasing crosslinking density. In comparison with the benchmark, all fabricated membranes have significantly higher conductivities than the AMX. Similarly to Khetsomphou et al. for hCEMs [12], the conductivities of the hAEMs were higher due to their higher apparent thickness compared to the reference. Slade et al. [32] suggested that the correlation between the thickness and conductivity of a membrane may be due to structural changes caused by a difference in water uptake, a layered structure of the membranes or production processes. 

### 3.2. Electrodialysis Experiments

#### 3.2.1. Limiting Current Density

Within the fabricated membranes, no statistically significant differences were observed with an average current value of 16.9 ± 0.3 mA.cm^−2^, which corresponds to a voltage of 19.2 ± 0.3 V (Table 3). The statistical analysis did not reveal any difference among the fabricated membranes compared to the AMX reference. As mentioned, the system was in a voltage-controlled mode and 13.0 V were imposed on the system for all demineralization. The value was chosen from previous experiments with hCEMs to ensure a better comparison of the system performances [12]. This value corresponded to 80% of the anode/cathode voltage difference associated with the lowest limiting current density found. In this study, 80% of the associated voltage with the lowest limiting current density would be 14.8 V. 

#### 3.2.2. Demineralization of Whey

**Figure 4:**
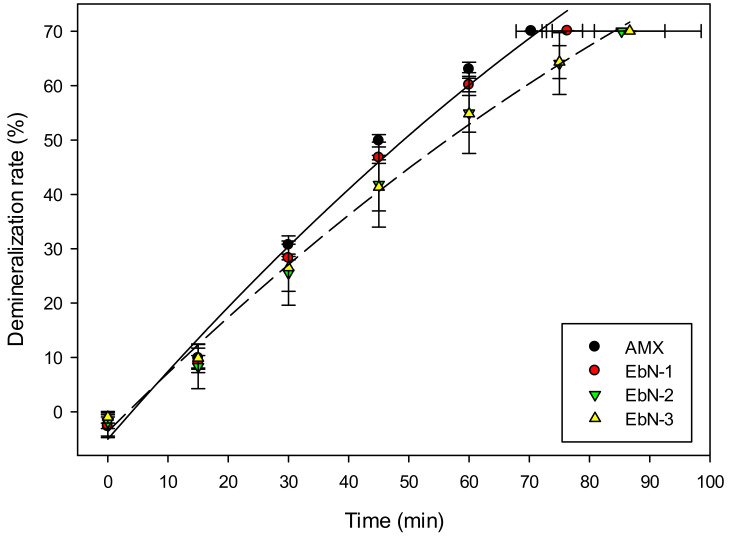
Whey demineralization evolution during ED with AMX or hAEMs.

To study the impact of hAEMs’ crosslinking density, their performances were tested in a whey demineralization process by electrodialysis. Figure 4 presents the demineralization rates as a function of time for each membrane. Even though the statistical analysis showed no difference within the fabricated membranes, the trend indicated that EbN-1 was different from EbN-2 and EbN-3 (76.3 ± 2.5 min vs. 86.0 ± 7.0 min). Compared to the reference (70.3 ± 2.5 min), EbN-2 and EbN-3 seemed to have a longer duration. The differences between EbN chemistries were not detected due to the high standard deviations obtained for EbN-2 (13.2 min). The three demineralization experiments performed in a back-to-back series with EbN-2 membrane, returned a progressively increased run time. This is likely due to fouling occurring on the surface or inside the membrane.

**Figure 5:**
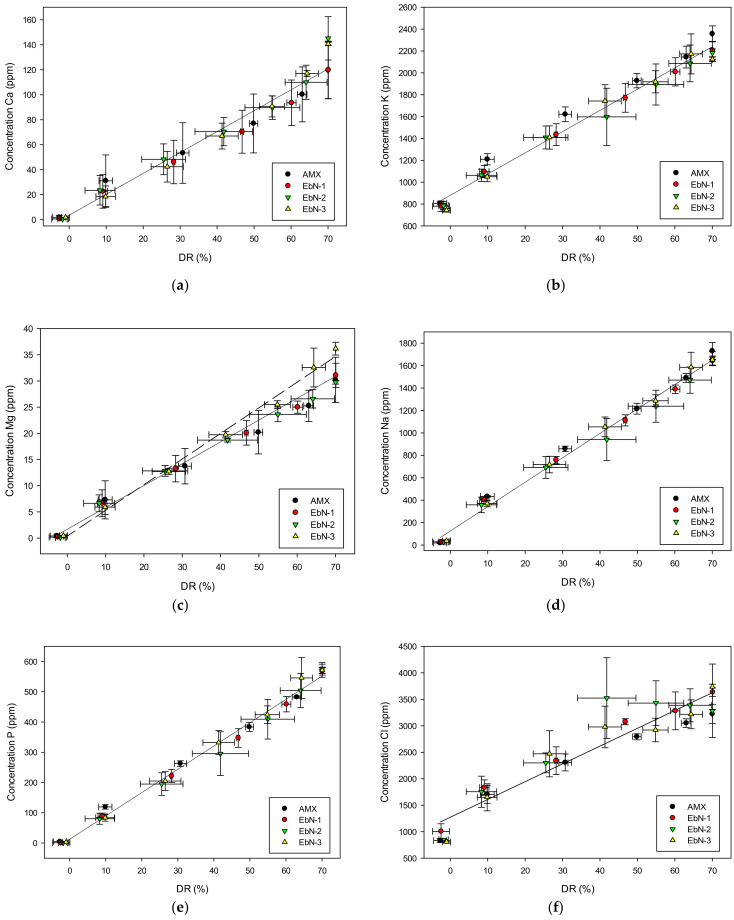
Ion concentration evolution in the KCl solution (concentrate) during ED according to the demineralization rate: (**a**) calcium; (**b**) potassium; (**c**) magnesium; (**d**) sodium; (**e**) phosphorous and (**f**) chloride.

The main ion concentrations in the KCl solution were analyzed throughout the process to confirm the mineralization/demineralization of the system. For all ions, their concentrations in the recovery compartment increased along with the demineralization rate as observed in Figure 5. Ions migrated linearly and at different speeds, which was explained by their intrinsic characteristics and their concentrations in the initial whey solution [33]. Hence, the first ions to migrate were potassium ones as they were the most abundant in the solution (55.6 ± 1.8% of the cations). It is also known that monovalent ions migrate faster than divalent ones since divalent ion hydration shells slow down their migration through the membrane due to a steric effect [34,35,36,37]. Overall, the membranes demonstrated similar migration for each ion except for magnesium ions. EbN-3 exhibited a faster migration of magnesium ions compared to the other membranes. This result might be due to the small concentration of magnesium in the feed solution. Moreover, the selectivity of ions by the IEMs is also governed by the affinity of ions with the resin. In general, divalent ions have a higher affinity with the resin than monovalent ones. This affinity is also emphasized by increasing the crosslinking density of the membrane [38].

#### 3.2.3. pH Variation

**Figure 6:**
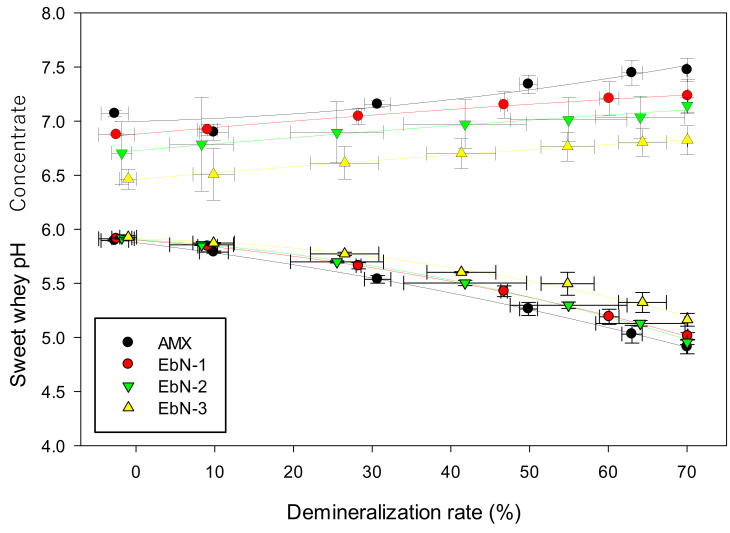
Evolution of pH in whey and KCl compartments during ED according to the demineralization with hAEMs or AMX.

Figure 6 represents the pH in both compartments of concentrate and diluate. Regarding the feed solution, the pH decreased by 1.0 unit all along the ED process for all membranes (around 5.9 at the beginning of the process and 4.9 at the end). Similar observations were made by Khetsomphou et al. with the tested hierarchical cation-exchange membranes [12]. This decrease, explained by Lemay et al. [39], is due to the transport of weak acids to the concentrate compartment. Indeed, during the process, the concentration of ions near the interface of the membrane decreases which leads to an amplification of the Donnan exclusion effect of H^+^ ions by the membrane. This increase in exclusion causes an increase in the membrane pH internal solution. Hence, when single charged ions such as H_2_PO_4_^−^ cross the membrane (with a high pH), the ions may dissociate into double-charged HPO_4_^2−^ to re-establish the equilibrium on the receiving side of the membrane. The H^+^ protons released from the transformation move to the diluate side leading to a decrease in pH [39]. With a 25% whey solution, decreases in pH by 0.39 and 1.38 pH units were also observed by Delbeke et al. for a 70% and 90% demineralization, respectively [40].

To make sure that the decrease in pH did not come from electrolytic water dissociation, the pH of the concentrate compartment was also tracked throughout the demineralization. The trend of a slight increase in the pH is similar for all membranes. No significant increase was noticed, indicating no water dissociation occurring in the system during the whole treatment and up to 70% demineralization. However, the initial pH, according to the membrane, was different. The results showed that pH (EbN-1) > pH (EbN-2) > pH (EbN-3). The trend indicated a decrease in pH with increasing crosslinking density. The slight acidification of the compartment would come from the precursors used which are slightly acidic. Commonly with acrylate resins, the acid groups of the coating react with water at the interface between the coating and the solution [41]. An acid–base reaction occurs leading to proton transfer and consequently to an acidification of the solution. The concentration of acrylate groups is higher for the hex-functional precursor and the concentration of this precursor increased with increasing crosslinking density, which explains the results obtained here.

#### 3.2.4. Global System Resistance

**Figure 7:**
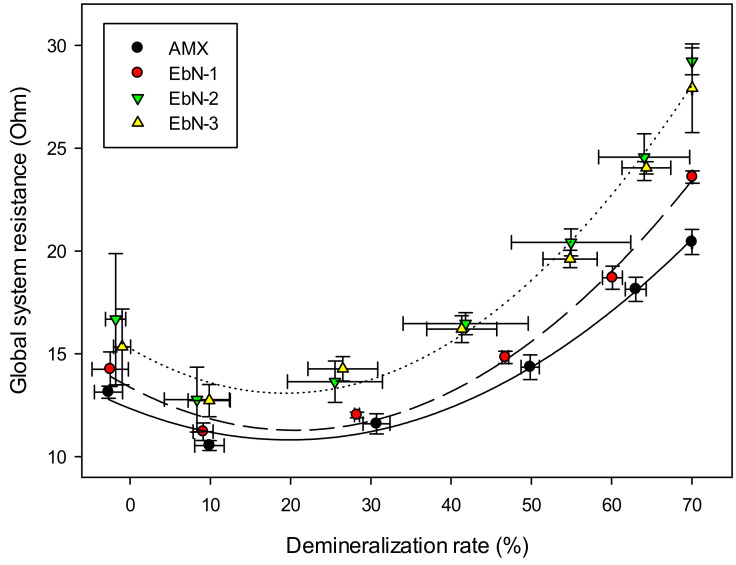
Global system resistance evolution during ED with hAEMs or AMX.

Figure 7 presents the global system resistance of the ED stack as a function of the demineralization rate. The parabola shape obtained here was explained by the concentrate and diluate conductivities. Similarly to the results acquired with the hCEMs, the conductivity of the compartment limits the electric charges transport. The least conductive solution impacts the evolution of the global system resistance [12]. Thus, when both solutions have similar conductivities, the global system resistance reaches a minimum as there is no more limiting compartment. Afterwards, a difference in conductivities can be observed between the diluate and concentrate compartments. Indeed, the diluate becomes the least conductive compartment, limiting the electric charges transports, hence leading to an increase in resistance. Regarding the performances of the membranes, the initial global system resistances were different. EbN-2 and EbN-3 exhibited higher global system resistance than EbN-1 and AMX. The membrane intrinsic conductivities also had an influence on the global system resistance performances. The comparison of the initial and final system resistance difference, showed as expected that EbN-2 and EbN-3 were significantly different from the benchmark (+64%). EbN-1 also demonstrated a higher global system resistance performance compared to AMX (+28%) but lower than those of the other EbN membranes. Overall, the fabricated hAEMs showed higher global system resistance than the reference, explained by the initial conductivities of the membranes. 

#### 3.2.5. Energy Consumption and Current Efficiency

Energy consumption was evaluated to assess the electrodialysis treatment performances (Table 4). The consumption of energy was the same for all of the membranes at 14.90 ± 0.30 Wh. 

The specific current efficiency was also calculated using potassium ions as it is the most abundant ion in the solution. The results presented in Table 4 demonstrate comparable performances for all membranes compared to the benchmark with an averaged current efficiency of 26.36 ± 1.79%. The current efficiencies obtained are lower than the ones previously obtained by Khetsomphou et al. on hCEMs [12]. These can be explained by the configuration of the ED stack and the compartment measured. Indeed, in the configuration used by Khetsomphou et al. [12], all cations migrate only in the KCl compartment. However, in the present study configuration, cations in the feed solution migrate in the KCl compartment and also in the rinsing solution compartment. Only the ions in the KCl compartment are measured, thus in this work not all of the migrated potassium was measured. Since, the concentration of potassium in the KCl compartment was used for the current efficiency calculation, lower values were consequently obtained here.

### 3.3. Change in Membrane Properties

**Figure 8:**
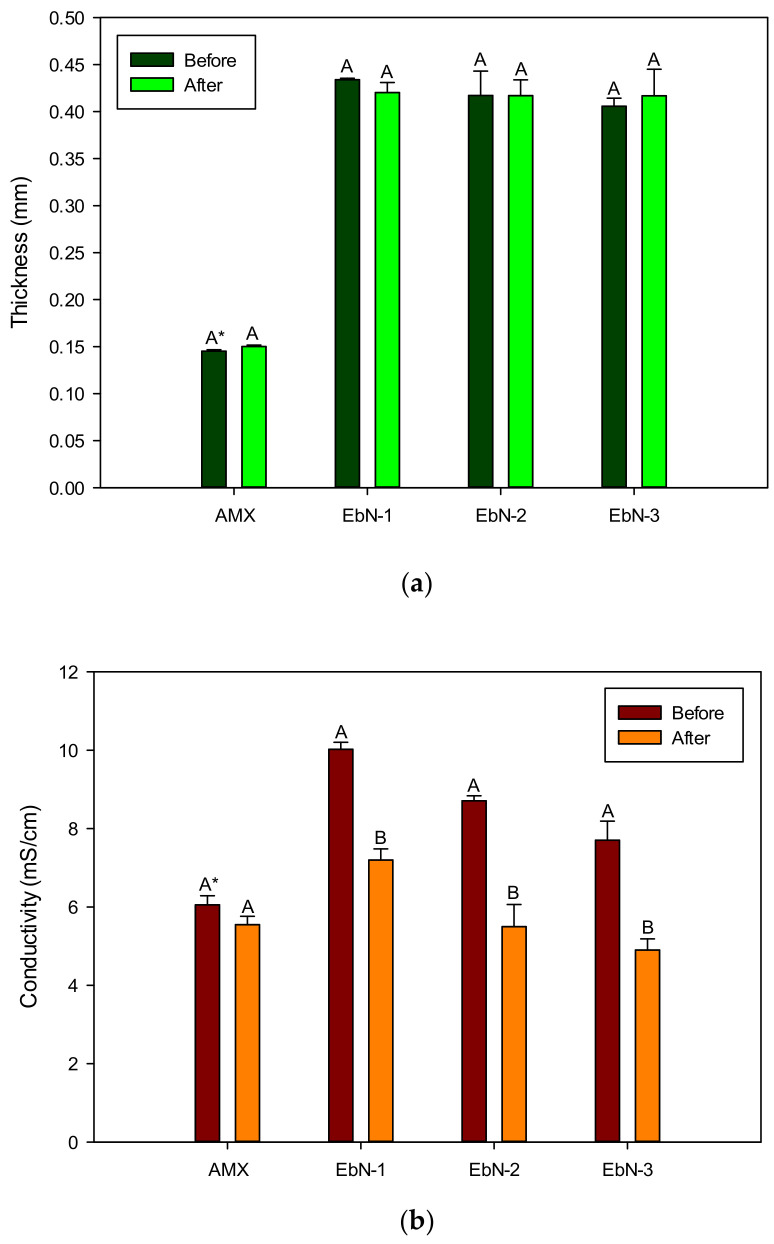

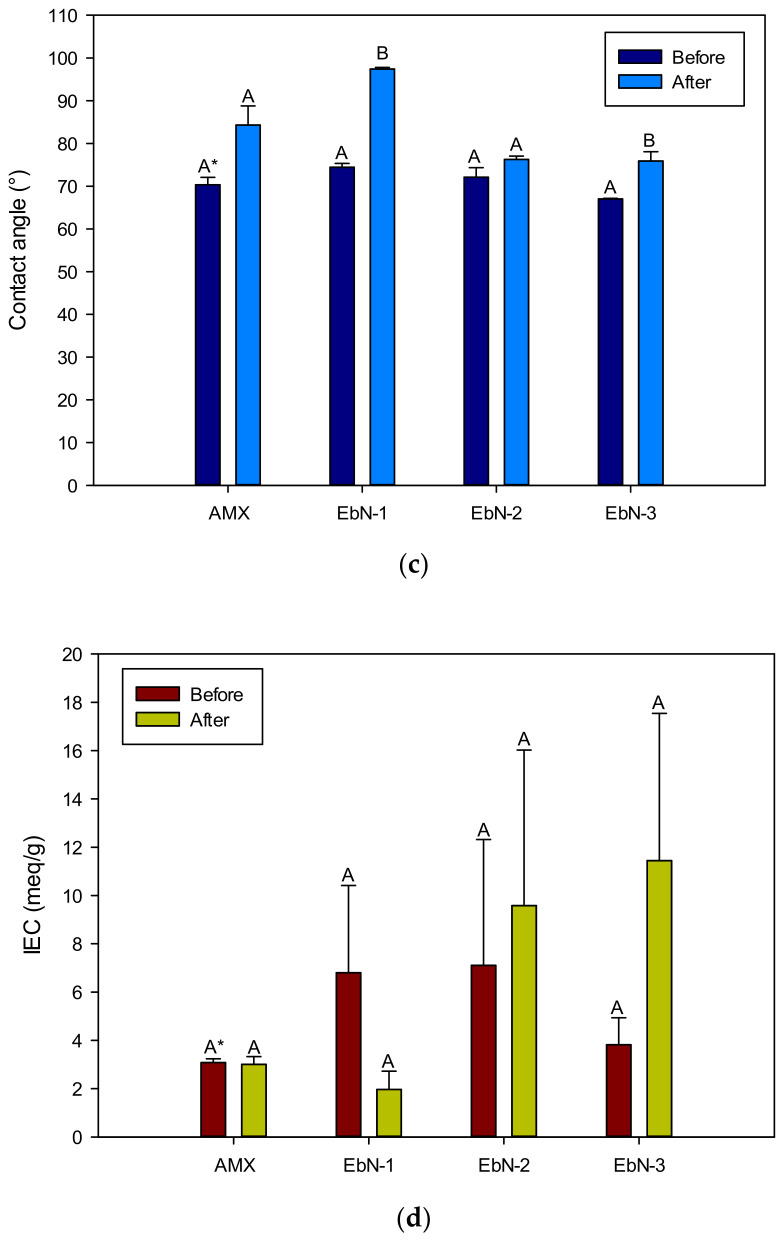
Thickness (**a**), conductivity (**b**), contact angle (**c**) and IEC (**d**) of hAEMs and AMX reported before and after three consecutive ED runs. * Values with different letters for the same membrane (A, B) before and after were significantly different at *p* < 0.05 (*t*-test).

The properties of the membranes were measured before and after electrodialysis to evaluate the impact of the process on the membranes. For the thicknesses of the membranes (Figure 8a), the results demonstrated no difference after the process for all membranes which indicates no significant fouling on the surface of the membranes. However, regarding the conductivity (Figure 8b) after electrodialysis, for all of the fabricated hAEMs, the conductivity decreased afterwards. The conductivity decreased by −28%, −37% and −36% for EbN-1, EbN-2 and EbN-3, respectively. These losses indicate a degradation of the hAEMs’ integrity throughout the process and that fouling may have occurred during the demineralization and inside the membrane. The obtained results can explain the higher global system resistance values obtained previously for EbN-2 and EbN-3 as well as the longer time needed to reach the 70% demineralization of the solution for the cited membranes.

Concerning the contact angle measurements (Figure 8c), the initial values ranging from 58° to 75° indicate a hydrophilic surface for all membranes. After ED, the values increased by +31% for EbN-1 and by +13% for EbN-3. No changes were observed for EbN-2. EbN-1 and EbN-3 membranes were demonstrated to be less hydrophilic after the experiments. Moreover, EbN-1 even demonstrated a value above 90° after the experiments, which results in the membrane being considered hydrophobic [42]. The surface of the membranes changed, which correlates with potential fouling occurring on the surface of the membranes but not affecting the thickness of the membrane. Regarding EbN-2, after dismantling the system, the surface of the membrane was not as smooth as before ED. Defects such as microcracks may have occurred during the experiment on the surface of the membrane. Those microcracks can lead to the infiltration of the water drop during the contact angle measurements, hence the still hydrophilic surface obtained [43,44]. The physical change in the surface endured by EbN-2 cannot exclude that fouling may have taken place on the surface of the membrane. 

Regarding the IEC (Figure 8d), the obtained standard deviations for all hAEMs were too great to detect differences before and after ED (5.91 ± 2.99 vs. 7.66 ± 5.74 meq/g, respectively) and also compared to the reference (3.08 ± 0.12 meq/g). Furthermore, regarding the AMX reference, the ion-exchange layer of the fabricated membranes is thin, around 10% of the whole membrane (between 38 and 52 µm, Figure 2), while the AMX membrane is 2.5 to 4 times thicker (145 µm, Table 2) than the ion-exchange layer of the hAEMs. Moreover, since the IEC value is based on the mass of dry membrane and the hAEMs’ porous substrate makes it denser, this needs to be taken into consideration when doing the experiments. Hence, IEC was also performed on the substrate/matrix alone and the obtained average value was 1.53 ± 0.04 meq/g. The presence of the substrate obscures the possibility to normalize the IEC value to membrane mass as only part of the membrane is constituted by an effective ion-exchange layer. This result indicates that the IEC values of the hAEMs obtained here are mainly governed by the combination of both coating and substrate. Indeed, during the sample preparation for the IEC determination, each sample was soaked in HCl overnight. Hence, an adsorption of H^+^ on silica surfaces in the porous substrate of hAEMs and difficulties in washing out the test solution from the substrate pores may cause great standard deviations in IEC results. Overall, this method does not appear to be representative of the hAEMs’ real ion-exchange capacity value and cannot truly be compared to the reference.

### 3.4. Fouling Assessment

#### 3.4.1. SEM-EDS

**Figure 9:**
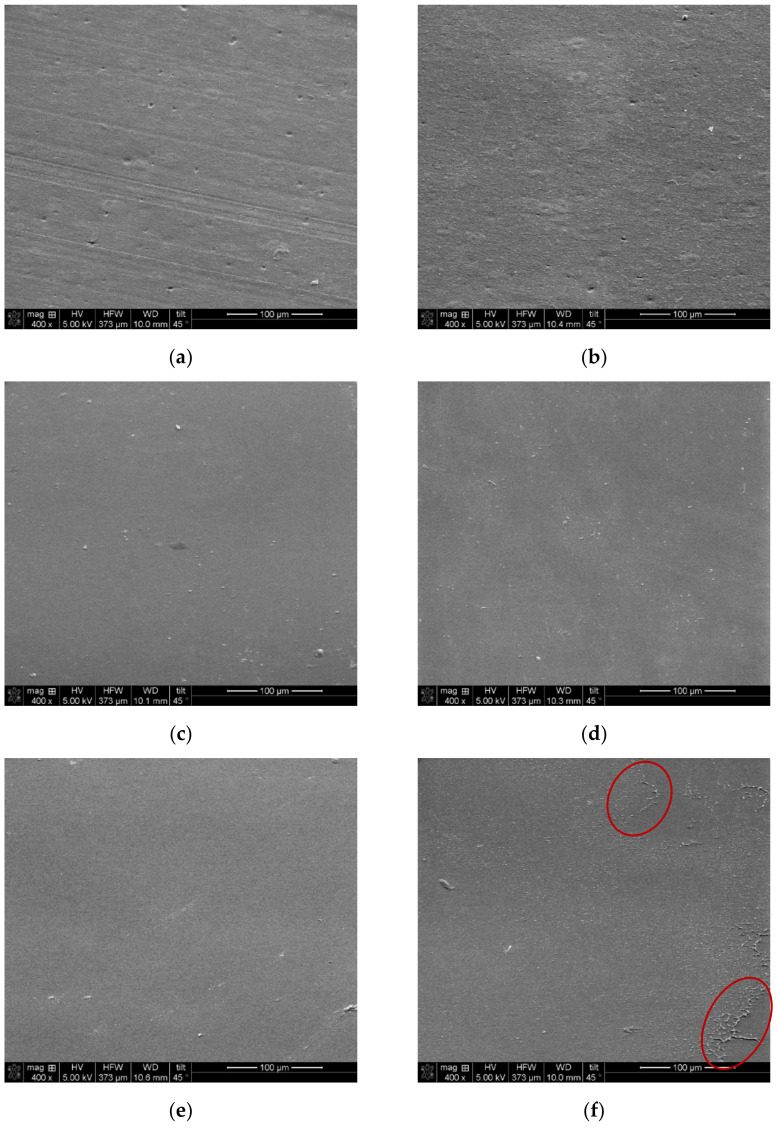

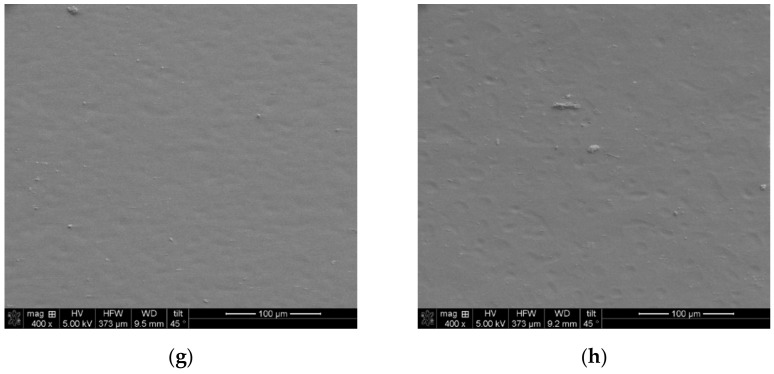
SEM images of the surface in contact with the whey solution of hAEMs and AMX before and after three consecutive ED runs: (**a**) AMX before, (**b**) AMX after, (**c**) EbN-1 before, (**d**) EbN-1 after, (**e**) EbN-2 before, (**f**) EbN-2 after, (**g**) EbN-3 before and (**h**) EbN-3 after.

To better understand the obtained performances in electrodialysis, fouling was investigated. SEM images of the surface side of the membranes in contact with the whey solution were taken before and after electrodialysis (Figure 9). Elemental analyses on the coated side were performed.

The SEM micrographs did not show any difference before and after ED for all membranes except EbN-2. For EbN-2, the membrane surface demonstrated defects after ED which explain the decrease in conductivity and the hydrophilicity results obtained above. Moreover, the elemental analysis (Table 5) did not reveal any statistical difference between a pristine membrane and a membrane after ED for any of the tested membranes. All membranes showed the presence of three main elements (C, O and Cl: 99% relative percentage). Similar results for clean similar AMX material have been reported [45]. The results indicate no scaling occurring on the surface of the membranes as minerals would have been detected by the elemental analysis. The images also indicate no fouling by proteins as the membrane surface would not have been visible. This result confirmed the ones obtained with the thicknesses of the membranes as no visual fouling was detected. However, organic fouling by smaller molecules can still occur and not be revealed by the elemental analysis. 

#### 3.4.2. ATR-FTIR

**Figure 10:**
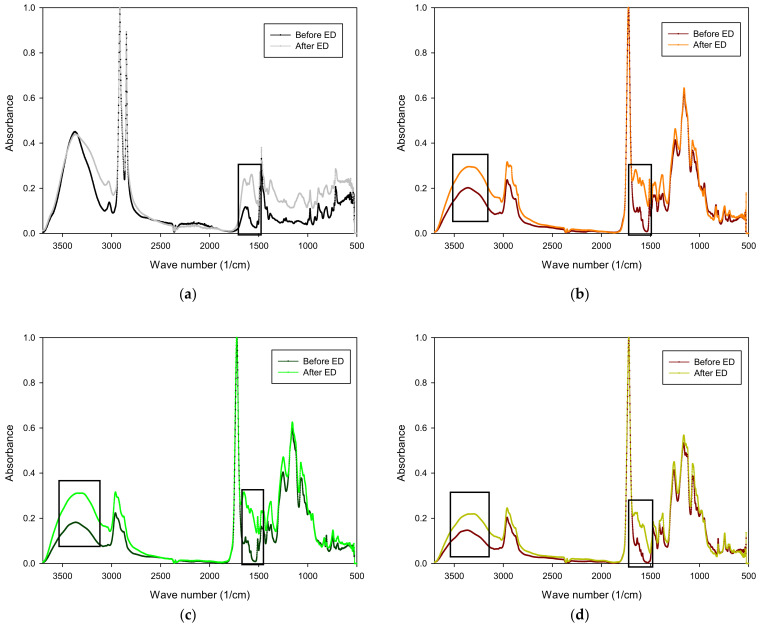
ATR-FTIR graphs of (**a**) AMX, (**b**) EbN-1, (**c**) EbN-2 and (**d**) EbN-3 before and after ED.

ATR-FTIR was carried out on the dilute side of the membrane before and after to assess if organic fouling occurred (Figure 10). Concerning the fabricated membranes, the spectra confirmed the presence of nitrogen compounds on the surface of the membranes after ED. Indeed, regions of 1600–1700 cm^−1^ and 1510–1580 cm^−1^ are related to the presence of amide I and amide II, respectively [31,46]. Vibrations around 1570–1580 cm^−1^ are associated with ionized carboxylic groups. Moreover, a difference in the spectra can be observed in the region around 1600 cm^−1^ corresponding to NH_3_^+^ groups coming from divalent ions of amino acids [18]. The results confirmed the presence of amide groups on the surface of the membranes. As no protein fouling was detected by the other analyses, the presence of amide groups is likely due to peptides and/or amino acids contained in the whey solution. Regarding the AMX reference, a difference can be observed in those same regions which also indicate the potential presence of peptides and/or amino acids. Persico et al. demonstrated that peptide fouling is mainly governed by electrostatic interactions [47]. Indeed, the accumulation and adsorption of negatively charged peptides and amino acids to neutralize the fixed charges present at the surface of the membranes lead to a decrease in the membrane conductivity [48]. However, electrostatic interactions are not the only mechanism responsible for peptides and amino acids fouling. Hydrogen bonds between functional groups plays a part in these foulants’ adsorptions [18]. EbN membranes contain carboxylic groups in the matrix. The higher the crosslinking, the higher the presence of carboxylic groups. Those groups can interact with peptides and amino acids which can explain the higher decrease in conductivity observed for the most crosslinked EbN membranes [22]. Regarding the AMX reference, these membranes contain benzene groups in the matrix which can interact with peptides and amino acids by hydrophobic interactions between aromatic groups [49]. 

The pristine hAEMs showed a peak around 3200–3400 cm^−1^ indicating strong O-H bond stretching. This peak is associated with a strong water peak or a carboxylic acid peak. The samples were dried for 5 days in a desiccator which is why association with the water peak was discarded. The pristine membranes contain O-H groups coming from the precursors. After ED, the used hAEMs demonstrated a broadening peak in this region. The whey solution contains lactic acid and citric acid; these acids can interact with the membranes when migrating through the membrane, decreasing the conductivity of the membrane [50,51,52]. Organic acids do not foul the membranes as they are too small. These negatively charged acids, like minerals, migrate towards the positive electrode by interactions with the fixed ions in the membrane. When the current is stopped, the migrating acids stay inside or on the surface of the membrane which explains their presence.

Overall, ATR-FTIR analyses revealed organic fouling occurring on the surface of the membranes by non-protein nitrogen, which explained the decrease in the conductivities of the membranes observed after ED and the reduced performances in ED compared to the reference AMX. Indeed, whey solutions contain non-protein nitrogen such as urea, arginine, glutamine, proline, etc. [53,54,55]. Arginine is also known to foul ion-exchange membranes [56,57]. Moreover, the analysis also revealed the presence of organic acids inside or on the surface of the membrane, which could also lead to the decrease in conductivity observed but these acids do not foul the inside or the surface of the membranes.

### 3.5. Global Performances

**Figure 11:**
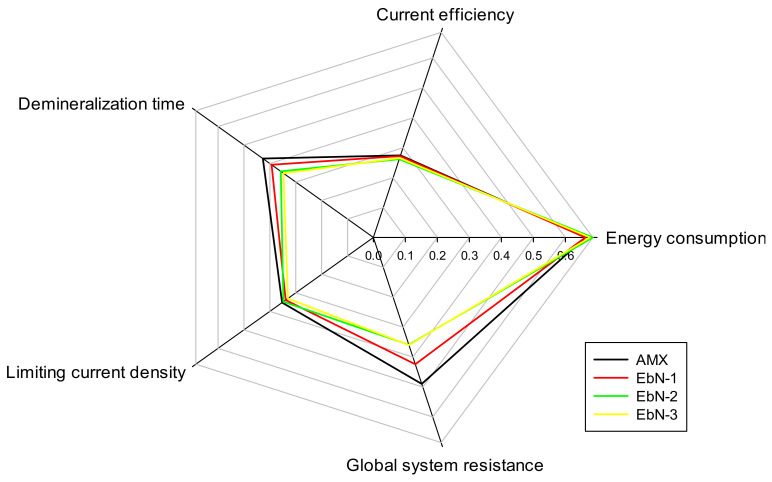
Evaluation of electrodialysis treatment performances using a radar graph.

Radar graphs (Figure 11) were plotted to assess and compare the performances of all of the membranes in ED on the same basis by evaluating the area obtained between the samples [12]. For each parameter, a high score corresponds to a good performance. The values of some parameters were obtained by taking the inverse of the value; hence, a bigger area was associated with a better ED performance. For example, for this study, a high score of global system resistance is associated with a high performance. These data were all recalculated to obtain a value score for all parameters within the same range [12].

Regarding the global membrane’s performances, the statistical analysis demonstrated that EbN-3 was different from the AMX. However, the trend indicated that EbN-2 was also different from the reference. The analysis did not pick up any difference, which may be due to the greater standard deviation for all parameters obtained with EbN-2. Indeed, the demineralization were run back-to-back, and EbN-2 may have endured a greater change in its integrity throughout the experiments. Overall, EbN-1 demonstrated similar global performances as the AMX reference.

Few other alternative membranes are reported in the literature for whey demineralization. Some alternative homogeneous and heterogeneous membrane performances in ED for relatively close or the same dairy product applications were studied (Table 6). Compared with alternative AEMs, the current efficiencies reported in the literature are higher, which can be explained by the way the calculations were done. The concentration of potassium was used in this study, whereas the concentration of all cations was used in the literature [33,58,59]. Regarding the energy consumption, the target demineralization rates were different (75%, 90% and 98%) as well as the initial solution (sweet whey concentrate, acid whey and evaporated sweet whey) and the configuration of the system (the size and number of cells) which explains the differences found here [60,61,62].

## 4. Conclusions

hAEMs were tested for the first time in the conditions commonly found for whey demineralization in the industry (18% (*w*/*w*) whey solution—70% demineralization). The impact of the coating formulation, in terms of crosslinking density, of the hAEMs on ED performances was also evaluated and the results compared to a commercial food grade reference AMX. All hierarchical membranes were able to demineralize 70% of an 18% whey solution with EbN-1 having a similar duration as the AMX control. Regarding the fabricated membranes, the crosslinking density of the coating had an impact on the membrane electrical conductivity values, also influencing the ED system resistance. An increase in crosslinking density led to an increased resistance. Moreover, the electrical conductivity values of the hAEMs decreased after the experiments which is likely due to organic fouling by peptides or amino acids. The ATR-FTIR results also demonstrated the presence of organic acids on the membrane surface which can also decrease the membrane electrical conductivity. These organic acids do not foul the inside or the surface of the membrane, and their presence is due to the electromigration of the species. Overall, all membranes demonstrated similar energy consumption and current efficiency as the benchmark AMX. Even though EbN-1 demonstrated a slight decrease in its properties, this membrane demonstrated the most similar performances as the reference. Consequently, this hAEM appears as the most promising one to be an alternative membrane to the commercial AMX for applications in the food industry.

However, more testing will be necessary to verify the compatibility with food streams of the hAEMs. As the dairy industry has cleaning protocols to clean the systems, the impact of cleaning solutions also needs to be investigated to consider those membranes as a potential alternative to food grade AMX membranes.

The next step would be to assemble the best fabricated hAEM, EbN-1, with the best hCEM from Khetsomphou et al. [12] in an ED system and compare the performances for whey demineralization with AMX and CMX commercial references. These experiments will bring the final proof for the use of these new alternative hIEMs in the food industry.

## Figures and Tables

**Table 1:** Ion-exchange coating formulation details.

Sample	EBE3105 *	EBE830
EbN-1	0.30	0.15
EbN-2	0.15	0.30
EbN-3	0.00	0.45

* EBE3105 and EBE830 are bi-functional and hex-functional UV-curable polyester acrylate resins, respectively.

**Table 2:** Thickness, Ra, Rz, conductivity and selectivity of hAEMs and commercial reference, and hAEM coating layer thickness.

Sample	Membrane Thickness (mm)	Coating Thickness (µm)	Ra (µm)	Rz (µm)	Conductivity (mS.cm^−1^)	Selectivity (%)
AMX	0.145 ± 0.002 ^b^*	-	0.26 ± 0.09 ^a^	3.43 ± 2.03 ^b^	6.06 ± 0.23 ^c^	89 ± 0 ^b^
EbN-1	0.434 ± 0.002 ^a^	36.59 ± 4.41 ^b^	0.55 ± 0.26 ^a^	5.80 ± 0.73 ^a,b^	10.03 ± 0.18 ^a^	86 ± 0 ^a^
EbN-2	0.417 ± 0.026 ^a^	45.48 ± 9.43 ^a,b^	0.59 ± 0.25 ^a^	10.31 ± 2.66 ^a^	8.71 ± 0.13 ^b^	91± 0 ^c^
EbN-3	0.406 ± 0.009 ^a^	54.34 ± 2.67 ^a^	0.29 ± 0.08 ^a^	9.31 ± 2.56 ^a^	7.71 ± 0.49 ^b^	94 ± 0 ^d^

* Values with different letters (a, b) for the same column were statistically significantly different (Tukey) at *p* < 0.05.

**Table 3:** ED cell limiting current densities and associated voltages for each membrane.

Sample	Limiting Current Density (mA.cm^−2^)	Associated Voltage (V)
AMX	17.6 ± 0.21 ^a,^*	18.46 ± 0.39 ^a^
EbN-1	16.9 ± 0.15 ^a^	18.61 ± 0.93 ^a^
EbN-2	17.4 ± 0.10 ^a^	19.31 ± 1.77 ^a^

* Values followed with different letters for the same column are statistically significantly different (Tukey) at *p* < 0.05.

**Table 4:** Consumption of energy and current efficiency during ED experiments with hAEMs or AMX.

Sample	Energy Consumption (Wh)	Current Efficiency (%)
AMX	15.10 ± 0.09 ^a,^*	27.45 ± 1.54 ^a^
EbN-1	15.10 ± 0.16 ^a^	25.25 ± 1.51 ^a^
EbN-2	14.60 ± 0.59 ^a^	26.19 ± 2.54 ^a^
EbN-3	14.78 ± 0.43 ^a^	26.56 ± 3.08 ^a^

* Values followed with different letters for the same column are statistically significantly different (Tukey) at *p* < 0.05.

**Table 5:** X-ray elemental analyses for the surface in contact with the diluate solutions for hAEMs and AMX before and after ED.

Element	AMX (At%)		EbN-1 (At%)		EbN-2 (At%)		EbN-3 (At%)	
	Before	After	Before	After	Before	After	Before	After
S	0.08	0.11	-	-	-	-	-	-
Cl	2.22	0.63	1.06	-	0.98	0.09	0.99	-
P	-	0.18	0.17	0.16	0.18	0.14	0.18	0.14
O	3.09	6.55	17.79	19.56	18.75	22.00	21.45	22.92
C	94.61	92.54	80.98	80.28	80.09	77.77	77.38	76.94

**Table 6:** Alternative AEM performances on dairy product applications found in the literature.

AEM (Manufacturer)	Solution	ED Unit	Duration (min)	Demineralization Rate (%)	Energy Consumption	Current Efficiency (%)	Reference
SA-1	WPC (10.0 wt. %)	10 cell pairs 100 cm^2^/membrane	60	64.2	640 kWh/eq removed	84.2	Pérez et al. [33]
Neosepta CMX—homogeneous (Astom)	1.50 L nanofiltered whey (18.0–20.0 wt. %)	8 cell pairs 37 cm^2^/membrane	260	90.0	26.5 kJ	70.0	Greiter et al. [58]
Neosepta AHA—homogeneous (Astom)	1.20 L acid whey (5.2 wt. %)	2 cell pairs 36 cm^2^/membrane	180	90.0	0.014 kWh/g	80.0–90.0	Chen et al. [59]
Neosepta AHA—homogeneous (Astom)	2.00 L sweet whey (6.5 wt. %)	2 cell pairs 36 cm^2^/membrane	180	75.0	5.9 kWh/ton of whey		Talebi et al. [60]
AEM-PES—heterogeneous (MemBrain)	30.00 kg acid whey (20.0% wt. %)	50 cell pairs 400 cm^2^/membrane	195	89.3	8.8 Wh/kg		Merkel et al. [62]
Ralex AM-PES TR I—heterogeneous (MEGA)	2.00 kg evaporated sweet whey (15.7 wt. %)	10 cell pairs 64 cm^2^/membrane	180	98.0	4.4 Wh/kg		Nielsen et al. [61]

## Data Availability

The raw data supporting the conclusions of this article will be made available by the authors on request.
